# Comparison of the effectiveness four years after Homo/Hetero prime-boost with 10 μg HP and 20 μg CHO recombinant hepatitis B vaccine at 1 and 6 months in maternal HBsAg-negative children

**DOI:** 10.3389/fimmu.2024.1308238

**Published:** 2024-04-10

**Authors:** Zhiying Yin, Tingcui Wen, Canya Fu, Junji Li, Quanjun Fang, Xiaoying Gong, Jialing You, Shuangqing Wang, Canjie Zheng

**Affiliations:** ^1^Department of Immunoprevention, Quzhou Center for Disease Control and Prevention, Quzhou, Zhejiang, China; ^2^School of Public Health, Zhejiang Chinese Medical University, Hangzhou, Zhejiang, China; ^3^Department of Microbiology, Quzhou Center for Disease Control and Prevention, Quzhou, Zhejiang, China

**Keywords:** recombinant hepatitis B vaccine, Hansenula polymorpha, Chinese hamster ovary cells, hepatitis B surface antibody, homo/hetero prime-boost

## Abstract

**Introduction:**

Limited data were available on the effectivenessfour years after Homo or Hetero prime-boost with 10 μg Hansenulapolymorpha recombinant hepatitis B vaccine (HepB-HP) and 20 μgChinese hamster ovary cell HepB (HepB-CHO).

**Methods:**

A crosssectional study was performed in maternalhepatitis B surface antigen (HBsAg)-negative children whoreceived one dose of 10 μg HepB-HP at birth, Homo or Heteroprime-boost with 10 μg HepB-HP and 20 μg HepB-CHO at 1 and 6months. HBsAg and hepatitis B surface antibody (anti-HBs) fouryears after immunization were quantitatively detected by achemiluminescent microparticle immunoassay (CMIA).

**Results:**

A total of 359 children were included; 119 childrenreceived two doses of 10 μg HepB-HP and 120 children receivedtwo doses of 20 μg HepB-CHO, called Homo prime-boost; 120children received Hetero prime-boost with 10 μg HepB-HP and 20μg HepB-CHO. All children were HBsAg negative. The geometricmean concentration (GMC) and overall seropositivity rate (SPR) ofanti-HBs were 59.47 (95%CI: 49.00 – 72.16) mIU/ml and 85.51%(307/359). Nearly 15% of the study subjects had an anti-HBsconcentration < 10 mIU/ml and 5.01% had an anti-HBsconcentration ≤ 2.5 mIU/ml. The GMC of the 20 μg CHO Homoprime-boost group [76.05 (95%CI: 54.97 – 105.19) mIU/ml] washigher than that of the 10 μg HP Homo group [45.86 (95%CI:31.94 – 65.84) mIU/ml] (*p* = 0.035). The GMCs of the Heteroprime-boost groups (10 μg HP-20 μg CHO and 20 μg CHO-10 μgHP) were 75.86 (95% CI: 48.98 – 107.15) mIU/ml and 43.65(95%CI: 27.54 – 69.18) mIU/ml, respectively (*p* = 0.041). Aftercontrolling for sex influence, the SPR of the 20 μg CHO Homoprime-boost group was 2.087 times than that of the 10 μg HPHomo group.

**Discussion:**

The HepB booster was not necessary in the generalchildren, Homo/Hetero prime-boost with 20 μg HepB-CHO wouldincrease the anti-HBs concentration four years after immunization,timely testing and improved knowledge about the self-pay vaccinewould be good for controlling hepatitis B.

## Introduction

Hepatitis B is caused by the hepatitis B virus (HBV), and chronic infection with HBV can lead to cirrhosis and liver cancer. The younger a person is infected, the greater the risk of developing chronicity. About 90% of children exposed to HBV early in life will eventually develop chronic hepatitis B (1). The World Health Organization (WHO) has called for the elimination of hepatitis by 2016, which depends on preventing new infections and reducing chronic infections (2). In 2022, approximately 6.4 million children under the age of 5 years worldwide will still have chronic HBV infection (3). Based on the dynamic simulation model, there will be 60 million people infected with HBV in China in 2030 and 10 million HBV-related deaths between 2015 and 2030 (4). HBV infection continues to be a serious public health problem. The hepatitis B vaccine (HepB) has been shown to be the most effective in protecting susceptible populations and preventing hepatitis B infection (5); an effective childhood vaccine against HBV has been available for more than 30 years (6). Three-dose HepB coverage reached 85% worldwide in 2019, but was about 30% in 2000 (7). According to the latest WHO estimates, chronic HBV infection in children under 5 years of age has declined from 5% in the pre-vaccine era to less than 1% in 2019 (8).

China included HepB in planning immunization in 1992 and national immunization program in 2002, and provided free vaccination to newborns since June 2005. The schedule consists of three doses to be given at birth, 1 month and 6 months of age. Three types of recombinant hepatitis B vaccine have been used in China, which were Hansenula polymorpha (HepB-HP), Saccharomyces cerevisiae (HepB-SC) and Chinese hamster ovary cells (HepB-CHO) (**Table 1**), and the dosages can be classified as 10 µg/dose, 20 µg/dose and 60 µg/dose (9). The Immunization Procedures and Instructions for Children of the Chinese Immunization Program (version 2021) recommended that 10 µg recombinant hepatitis B vaccines can be used for neonatal immunization, but HepB-CHO dosage increased by 20 µg for newborn with HBsAg positive mother (10). The vaccine confidence crisis in 2013 led to a shortage of HepB in China. To reduce the impact of the crisis, the Chinese Center for Disease Control and Prevention proposed that 10 μg HepB-HP, 10 μg HepB-SC, 10 μg and 20 μg HepB-CHO could be used interchangeably in routine immunization. Sequential immunization with 10 μg HepB-HP, 10 μg HepB-SC and 20 μg HepB-CHO has shown good immunogenicity and safety in previous studies (11).

**Table 1:** Comparison of the components and properties of three types of HepB.

Components	HepB-CHO	HepB-HP	HepB-SC
Active ingredient	hepatitis B surface antigen (HBsAg)	HBsAg	HBsAg
HBsAg derived from	mammalian cell (Chinese hamster ovary)	yeast cell (Hansenula polymorpha)	yeast cell (Saccharomyces cerevisiae)
Adjuvant	aluminum hydroxide [Al(OH)3], Sodium chloride	Al(OH)3, Sodium chloride	Al(OH)3, Sodium chloride
Dosage	HBsAg 10μg/0.5 ml, HBsAg 20μg/1.0 ml	HBsAg 10μg/0.5 ml, HBsAg 20μg/0.5 ml	HBsAg 10μg/0.5 ml, HBsAg 20μg/1.0 ml, HBsAg 60μg/1.0 ml
Usage	10μg and 20μg both used for people of all ages	10μg used for people of all ages, 20μg used for people over 16 years old	10μg used for people under 16 years old, 20μg and 60μg both used for people over 16 years old

Hepatitis B surface antibody (anti-HBs) is a protective antibody of the human body that can protect the body from HBV infection. The concentration of anti-HBs below 10 mIU/ml is considered the threshold for effective protection against HBV infection (12). Anti-HBs levels in children with maternal HBsAg positivity declined sharply from 7 months to 2 years of age, suggesting that a HepB booster should be given before 2 years of age (13). Monitoring the anti-HBs levels after primary vaccination and timely administration of a booster dose are important. However, it was unknown how antibody levels in maternal HBsAg-negative children changed after primary immunization, whether a booster was needed, or when the booster was given. In this study, we compared the effectiveness four years after Homo or Hetero prime-boost with 10 μg HP and 20 μg CHO HepB at 1 and 6 months in maternal HBsAg-negative children, explored whether the anti-HBs levels could be improved by different dosages and types of HepB.

## Materials and methods

### Study setting

Quzhou is a prefecture-level city located in the west of Zhejiang Province, East China, with two districts, three counties and one county-level city. It covers an area of 8,844 square kilometers and had a population of 2.29 million at the end of 2022. The immunization of local children was implemented through 102 vaccination clinics in Quzhou. The first dose of HepB is given at birth in the hospital maternity ward according to national immunization procedures. The HepB used in hospital maternity wards was freely provided by the Zhejiang Center for Disease Control and Prevention. For neonatal HepB, 10μg HepB-HP was always used free of charge in Quzhou from 2010 to 2020, while the use of 20 μg HepB-CHO was limited by self-pay. Basic information, including name, sex, birth date, birth weight and maternal hepatitis B infection status, and vaccination information, including vaccination date, vaccine type, dosage and manufacturer has been recorded in the Zhejiang Provincial Immunization Information System (ZJIIS) since 2005. Each person has a unique bar code, and the clinic doctors record the vaccination information into the ZJIIS. The Chinese Adverse Events Following Immunization (AEFI) Information Management System was launched in 2005 and was designed to record post-vaccination adverse events that suspected vaccination-related.

### Study design and participants

**Figure 1:**
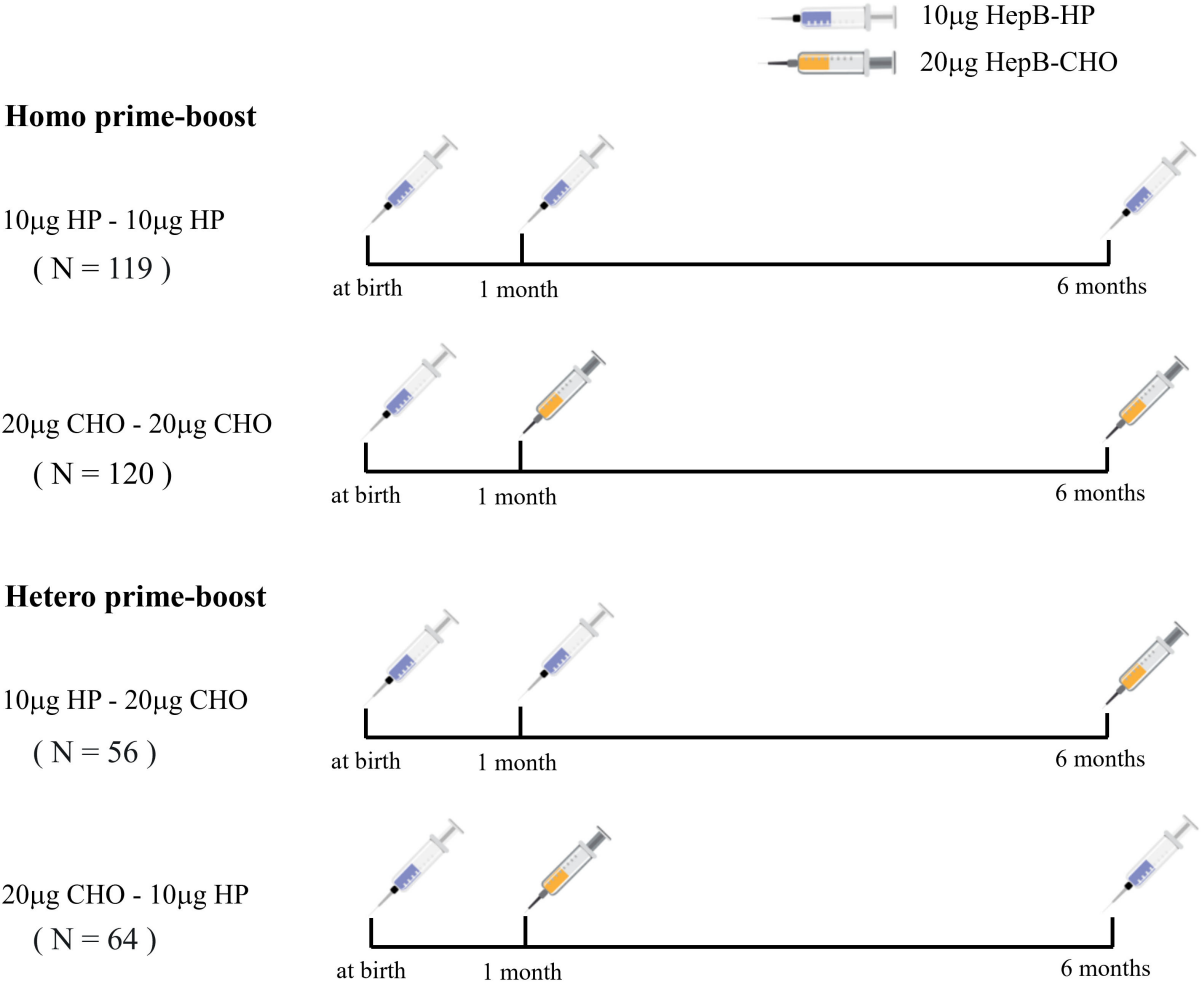
The schedules for Homo and Hetero prime-boost with 10 μg HP and 20 μg CHO recombinant hepatitis B vaccine.

This cross-sectional study was conducted in Kecheng and Qujiang District of Quzhou from May to August 2022. Children who were born between 1 January 2017 and 30 June 2018, and received one dose of 10 μg HepB-HP at birth, Homo or Hetero prime-boost with 10 μg HepB-HP and 20 μg HepB-CHO at 1 and 6 months were included in our study. Homo prime-boost with two doses of 10 μg HepB-HP or 20 μg HepB-CHO, named the 10 μg HP-10 μg HP group and 20 μg CHO-20 μg CHO group, respectively. Hetero prime-boost with one dose of 10 μg HepB-HP at 1 month and 20 μg HepB-CHO at 6 months was called the 10 μg HP-20 μg CHO group, the reverse was defined as the 20 μg CHO-10 μg HP group (**Figure 1**). Children with severe disease, HBsAg-positive mother, not participating in the 0-1-6 program, or with incomplete or unknown vaccination records were excluded. Parents were informed by telephone about the purpose and significance of the study. Questionnaires and blood samples were taken from the subjects. The questionnaires, including sociodemographic information, health status, HepB vaccination history and maternal hepatitis B infection status, were administered by the investigators at the vaccination clinic, and abnormal immune history was checked with the information system. Written informed consent was obtained from a guardian of each subject, and the test results were communicated separately to the guardian. The Chinese AEFI Information Management System was used to reconcile the post-immunization side effects of HepB for each subject.

The sample size was estimated using a non-inferiority design method with the following formula: N = $\frac{{\left(Z_{\text{α}}\sqrt{2\bar{pq}}+Z_{\text{β}}\sqrt{p_{0}q_{0}+p_{1}q_{1}}\right)}^{2}}{{\left(p_{1}-p_{0}\right)}^{2}}$ , where $p_{1}\;$ and $p_{0}$ represent the expected positive rate of anti-HBs in the 20 μg CHO-20 μg CHO and 10 μg HP-10 μg HP groups, respectively, $\bar{p}$ is the mean of the two expected positive rates, *q* = 1- *p* and both $Z_{\text{α}}$ and $Z_{\text{β}}$ are areas of normal distribution areas. Combined with previously reported seropositivity rates of three doses of 10 μg HepB-HP and 20 μg HepB-CHO at different times after immunization, α = 0.05, β = 0.2, $p_{0}$ = 60%, $p_{1}$ = 80%, and the expected sample size was 108 for each group. A total of 373 children were recruited, excluding 3 children who did not participate in the 0-1-6 program and 3 children with unknown immune history in the 10 μg HP-10 μg HP group, 5 children in the 10 μg HP-20 μg CHO group and 3 in the 20 μg CHO-20 μg CHO group with HBsAg-positive mother. Finally, 359 eligible children were enrolled in this study.

### Specimen collection and testing

Next, 3 ml of venous blood samples was collected from each participant. The serum was separated and placed in a screw-capped centrifuge tube, transferred to a freezer box and stored in a refrigerator below −20°C. HBsAg and Anti-HBs was detected quantitatively using a chemiluminescent microparticle immunoassay (CMIA) by the laboratory of the Quzhou Center for Disease Control and Prevention. The kits used in our study were developed by Shenzhen Yahuilong Biotechnology CO., LTD (lot number: 20220601; validity period: 20230725), the detection ranges of the anti-HBs and HBsAg assay measured using Shenzhen Yahuilong iFlash 3000 analyzer were 0 ~ 1000.00 million international units per milliliter (mIU/ml) and 0 ~ 250.00 international units per milliliter (IU/ml), respectively; samples that exceed the upper limit of the range can be retested with appropriate dilutions to obtain a result close to the true concentration. The kit calibrations of the anti-HBs and HBsAg were traceable to the WHO 07/164 Anti-hepatitis B immunoglobulin international standard and the WHO 00/588 Hepatitis B surface antigen international standard. The cutoff value of anti-HBs was 10 mIU/ml, the concentrations ≥ 10 mIU/ml was defined as positive and the reverse was negative; the cutoff value of HBsAg was 0.05 IU/ml, sample concentrations <0.05 IU/ml were considered negative and the opposite was positive. The 10 μg HepB-HP and 20 μg HepB-CHO vaccines were manufactured by Dalian Hanxin Biopharmaceutical CO., LTD and North China Pharmaceutical Golden Biotechnology CO., LTD, respectively.

### Statistical analysis

All data were double entered into Epidata 3.1 and exported to Microsoft Office Excel (version 2010) for analysis. The seropositive rate (SPR) of anti-HBs, the geometric mean concentrations (GMCs) and their 95% confidence intervals (CIs) were calculated, and anti-HBs concentrations were logarithmically transformed for evaluation. Reverse cumulative distribution curves were used to show the proportional distribution of antibody concentrations in each group. Post-vaccination time was defined as the interval between blood collection and completion of the primary vaccination. The t-test was used to compare the GMCs of the groups, and chi-squared was used to calculate the difference in SPRs. Cochran’s and Mantel-Haenszel statistics was used to calculate χ^2^_MH_ and Relative Risk (RR) to eliminate confounding influence. Statistical analyses were performed using SPSS for Windows (version 16.0; SPSS Inc., USA). All *p* values are two-tailed, and the value of *p* < 0.05 was defined as statistically significant.

## Results

### Sociodemographic characteristics

A total of 359 children (181 boys and 178 girls) were included in the study; the number of participants in the 10 μg HP-10 μg HP, 20 μg CHO-20 μg CHO, 10 μg HP-20 μg CHO, 20 μg CHO-10 μg HP groups were 119 (57/62), 120 (58/62), 56 (33/23) and 64 (33/31), the sex ratio in the Homo prime-boost group (χ^2 ^= 0.005, *p* = 0.946) and in the Hetero prime-boost group (χ^2^ = 0.655, *p* = 0.418) were both no statistically significant difference. The mean ages of the children between the Homo prime-boost groups (10 μg HP-10 μg HP vs 20 μg CHO-20 μg CHO) were 4.50 (95%CI: 4.44 – 4.56) and 4.51 (95%CI: 4.41 – 4.61) years (*t* = 1.546, *p* = 0.123), and the mean age between the Hetero prime-boost groups (10 μg HP-20 μg CHO vs 20 μg CHO-10 μg HP) were 4.54 (95%CI: 4.44 – 4.64) and 4.51 (95%CI: 4.41 – 4.61) years (*t* = 0.433, *p* = 0.666). The time after primary vaccination between the Homo prime-boost groups were 3.92 (95%CI: 3.83 – 4.01) and 3.93 (95%CI: 3.82 – 4.04) years (*t* = 1.482, *p* = 0.140), and the time after immunization between the Hetero prime-boost groups was also no statistically significant difference (*t* = 1.103, *p* = 0.272). Of the 359 subjects, 215 children were from Kecheng District and 144 children were from Qujiang District, and 85.83% (206/240) children received 20μg HepB-CHO at the Kecheng vaccination clinic, whereas only 14.17% (34/240) children received 20μg HepB-CHO at the Qujiang vaccination clinic. All children with HBsAg-negative mother in the study received the first dose of 10μg HepB-HP at birth and completed the full primary vaccination on 186.3 (95% CI: 183.4 – 189.6) days after birth. At the time of the survey, none of the 359 children had been identified in the Chinese AEFI Information Management System as having experienced HepB-related adverse events.

### GMCs of HBsAg and anti-HBs

**Figure 2:**
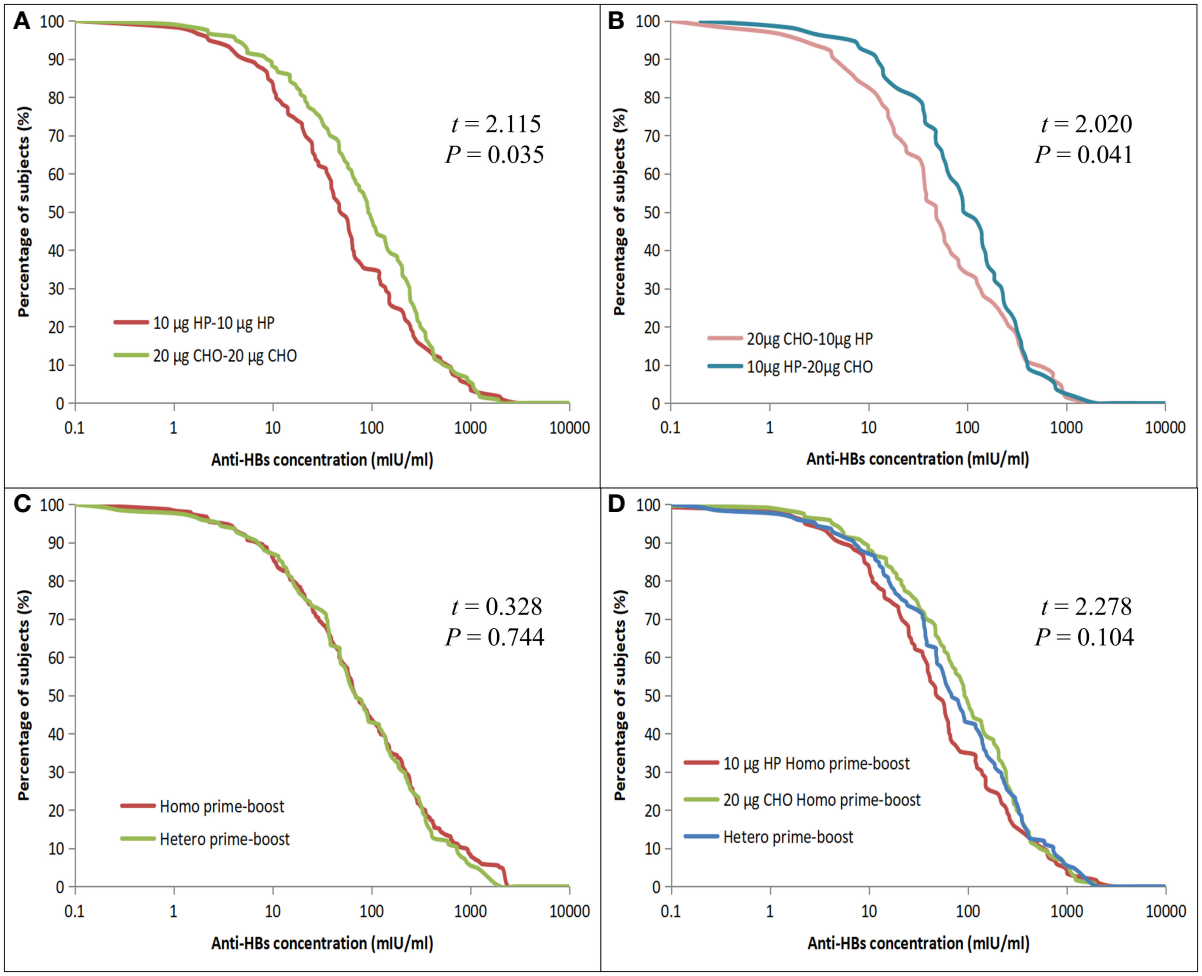
Reverse cumulative distribution curves for individual anti-HBs concentrations in the Homo and Hetero prime-boost groups. **(A)** Comparison of the 10 μg HP and 20 μg CHO for Homo prime-boost groups. **(B)** Comparison of the 20 μg CHO-10 μg HP and 10 μg HP-20 μg CHO for Hetero prime-boost groups. **(C)** Comparison of the Homo and Hetero prime-boost groups. **(D)** Comparison of the 10 μg HP, 20 μg CHO Homo and Hetero prime-boost groups.

All 359 children had an HBsAg concentration of <0.05 IU/ml. The GMC and 95% CI of anti-HBs after four years of primary vaccination was 59.47 (95%CI: 49.00 – 72.16) mIU/ml, the anti-HBs concentrations < 10 mIU/ml and ≤ 2.5 mIU/ml accounted for 14.48% (52/359) and 5.01% (18/359), respectively. The anti-HBs concentration of the 20 μg CHO-20 μg CHO group [76.05 (95%CI: 54.97 – 105.19) mIU/ml] was higher than that of the 10 μg HP-10 μg HP group [45.86 (95%CI: 31.94 – 65.84) mIU/ml] (*t* = 2.115, *p* = 0.035) in the Homo prime-boost vaccination. When the anti-HBs concentration was less than 1 mIU/ml or more than 430 mIU/ml, the percentages of anti-HBs concentration between the Homo groups were almost equal; but when the anti-HBs concentration was between 1 mIU/ml and 430 mIU/ml, the percentages of the 10 μg HP-10 μg HP group decreased more rapidly than that of the 20 μg CHO-20 μg CHO group (**Figure 2A**). The anti-HBs concentration of the 10 μg HP-20 μg CHO group [75.86 (95%CI: 48.98 – 107.15) mIU/ml] was also higher than that of the 20 μg CHO-10 μg HP group [43.65 (95%CI: 27.54 – 69.18) mIU/ml] (*t* = 2.020, *p* = 0.041) in the Hetero prime-boost vaccination. When the anti-HBs concentration was ≥ 328 mIU/ml, the percentages of the 20 μg CHO-10 μg HP group decreased faster than that of the 10 μg HP-20 μg CHO group (**Figure 2B**). The anti-HBs concentration between Homo prime-boost and Hetero prime-boost was no statistically significant difference (**Figure 2C**), and the difference among two Homo prime-boost and Hetero prime-boost was also no statistically significant difference (**Figure 2D**).

### Seropositive rate of HBsAg and anti-HBs

All 359 children were HBsAg negative. The overall seropositive rate of anti-HBs four years after primary vaccination was 85.51%(307/359), and the SPRs of Homo and Hetero prime-boost were 85.35% and 85.83% (*χ*2 = 0.015, *p* = 0.903). The SPRs of the 10 μg HP and 20 μg CHO Homo prime-boost group were 83.19% and 87.50% (*χ*2 = 0.887, *p* = 0.346). After controlling for sex influence, the SPR of the 20 μg CHO Homo prime-boost group was 2. 087 times than that of the 10 μg HP Homo prime-boost group (χ^2^_MH_=4.026, *p* =0.045, RR = 2.087). There was no significant difference in SPRs between boys and girls or between 3~ and 4~ years after vaccination in the Homo prime-boost groups. The SPRs in the Hetero prime-boost groups (10 μg HP-20 μg CHO vs 20 μg CHO-10 μg HP) was no statistically significant difference (*χ*2 = 2.369, *p* = 0.124). The difference between the Hetero prime-boost groups was also not statistically significant even eliminating the confounding factors of gender and time after vaccination (**Table 2**). The percentages of the anti-HBs concentrations ≤ 2.5 mIU/ml and < 10 mIU/ml were 5.01% and 14.48%. The proportions of anti-HBs concentrations ≤ 2.5 mIU/ml in Homo and Hetero prime-boost were 5.02% and 5.00%, and the proportions of anti-HBs concentrations < 10 mIU/ml in Homo and Hetero prime-boost were 14.64% and 14.17% (**Table 3**).

**Table 2:** The seropositive rate of anti-HBs in the Homo and Hetero prime-boost groups.

Group	Total	Gender				Time after vaccination			
		Boy	Girl	*χ*2	*p*	3~4 years	4~5 years	*χ*2	*p*
**Homo prime-boost**	239	115	124	4.026	0.045	138	101	0.536	0.464
10 μg HP-10 μg HP	119	57	62	0.043	0.837	74	45	0.624	0.429
Positive	99	47	52			60	39		
SPR(%)	83.19	82.46	83.87			81.08	86.67		
20 μg CHO-20 μg CHO	120	58	62	1.545	0.214	64	56	0.306	0.580
Positive	105	53	52			57	48		
SPR(%)	87.50	91.38	83.87			89.06	85.71		
*χ*2	0.887								
*p*	0.346								
**Hetero prime-boost**	120	66	54	1.787	0.181	72	48	1.600	0.206
10 μg HP-20 μg CHO	56	33	23	0.278	0.598	34	22	0.198	0.656
Positive	51	29	22			30	21		
SPR(%)	91.07	87.88	95.65			88.23	95.45		
20 μg CHO -10 μg HP	64	33	31	0.271	0.603	38	26	0.166	0.684
Positive	52	26	26			32	20		
SPR(%)	81.25	78.78	83.87			84.21	76.92		
*χ*2	2.369								
*p*	0.124								

**Table 3:** Distribution of the anti-HBs concentrations in the Homo and Hetero prime-boost groups.

Anti-HBs (mIU/ml)	No.	%	Hetero prime-boost				Hetero prime-boost			
			10 μg HP-10 μg HP		20 μg CHO-20 μg CHO		10 μg HP-20 μg CHO		20 μg CHO -10 μg HP	
			No.	%	No.	%	No.	%	No.	%
≤ 2.5	18	5.01	7	5.88%	5	4.17%	2	3.57%	4	6.25%
< 10	52	14.48	20	16.81%	15	12.50%	5	8.93%	12	18.75%
10~100	161	44.85	58	48.74%	48	40.00%	24	42.86%	31	48.44%
100~1000	134	37.32	36	30.25%	52	43.33%	26	46.43%	20	31.25%
≥ 1000	12	3.34	5	4.20%	5	4.17%	1	1.79%	1	1.56%

## Discussion

The introduction of hepatitis B vaccination at birth or in infancy prevents pediatric HBV infection and is cost effective or even cost saving (14). However, the long-term persistence of immunogenicity decreases with increasing age after vaccination (15). The GMC and SPR of anti-HBs after vaccination are important indicators to assess the immunogenicity of HepB. In our study, a GMC of 59.47 (95%CI: 49.00-72.16) mIU/ml with an SPR of 85.51% was observed, showing good immunogenicity in children who had completed the full primary course of HepB vaccination four years ago. A number of studies have shown that the persistence of anti-HBs is related to a variety of factors, including the mother’s carrying status, the regimen and type of vaccine used, the timeliness of the first dose of vaccine, and the level of anti-HBs at the end of primary vaccination (16, 17). The concentration of anti-HBs can be conferred by vaccination and/or natural infection. HBV is mainly transmitted sexually and can also be passed from mother to child; children aged 4.5 years with HBsAg-negative mothers usually have a lower risk of exposure to HBV. Thus, natural infection did not contribute too much in our study and the level of anti-HBs was mainly related to vaccine immunity. The GMC of 76.05 (95%CI: 54.97 - 105.19) mIU/ml in the 20 μg CHO Homo prime-boost group was higher than that of 45.86 (95%CI: 31.94 - 65.84) mIU/ml in the 10 μg HP Homo prime-boost group (p = 0.035), the main difference between the two groups being the type and dosage of vaccine used for the prime-boost, which was similar to the difference in GMCs at 1 month after primary vaccination in the study by Xia Wei et al (18).

Apart from the difference in dosage between 10μg HepB-HP and 20μg HepB-CHO, the main difference was the source of HBsAg. The HBsAg of HepB-HP and HepB-CHO was derived from Hansenula polymorpha yeast cells and Chinese hamster ovary cells, respectively. Chinese hamster ovary cell-derived HepB is a mammalian cell line, recombinant HBsAg derived from this cell line is characterized by a spatial structure similar to wild virus (19), and the molecular structure was closer to native HBsAg than that of HP yeast cells. In addition, the gene sequence of CHO-derived HBsAg was encoded by pre-S1, pre-S2 and S envelope protein, whereas HP-derived HBsAg was encoded by the S region. The higher immunogenicity of CHO-derived HBsAg is related to its gene sequence, molecular weight, molecular size and number of HBsAg monomers (20). Higher antibody concentrations and SPRs were found with 3A-HepB containing 3 HBV surface antigens, pre-S1, pre-S2 and S, than with 1A-HepB containing only the small S antigen in adults aged 18-45 years (21), 3A-HepB has been recommended for the prevention of HBV infection in adults, including those with stable and controlled chronic disease (22). The adjuvant was a substance that could non-specifically alter or enhance the body’s specific immune response to antigens and act as a supporting actor. The most commonly used adjuvant was Al(OH)3, Cysteine p Guanine Oligodeoxynucleotide (CpG-ODN) is a new adjuvant that has been developed in recent years. A recent study showed that CpG-ODN induced a higher antibody response compared to Al(OH)3 (23). The adjuvants for 10 μg HepB-HP and 20 μg HepB-CHO were the same in our study, which were mainly Al(OH)3 and sodium chloride. The effect of 20μg HepB-CHO was superior to that of 20 μg HepB-SC (24). The production cost for 20μg HepB-CHO was expensive and limited its use in China. **Figure 2B** shows that the GMCs between the heterologous prime-boost groups (10 μg HP-20 μg CHO vs 20 μg CHO-10 μg HP) were 75.857 (95%CI: 48.98 – 107.15) mIU/ml and [43.65 (95%CI: 27.54 – 69.18) mIU/ml] (*p* = 0.041), the heterologous 10 μg HP-20 μg CHO prime-boost schedule appeared to be superior to the 20 μg CHO-10 μg HP schedule, requires further investigation in the future.

In our study, more than 85% of children had anti-HBs levels ≥ 10 mIU/ml four years after primary vaccination, with almost 15% of the study sample being unprotected. HepB non-responders or a decline in immunity to unprotective levels may explain the lack of protective antibodies, but the reason could not be determined in our study due to the lack of baseline antibody levels after the full course of primary immunization. Our study also showed that 5.01% (18/359) of subjects had anti-HBs concentrations ≤ 2.5 mIU/ml, and a study in China suggested that the factor of anti-HBs concentration < 2.5 mIU/ml may be a greater risk of breakthrough infection (25). Although a study in the United States showed that booster doses were not needed in the general population 35 years after primary immunization with HepB (26), timely testing and decisions about booster immunization based on antibody levels were still very important. The proportions of anti-HBs concentration (100 ≤ anti-HBs < 1000 mIU/ml) in the 10 μg HP-10 μg HP and 20 μg CHO-20 μg CHO groups gradually increased, further demonstrating that the antibody levels of anti-HBs derived from 20μg HepB-CHO were superior to those from 10μg HepB-HP, consistent with the above study. Research has shown that working on attitudes and knowledge about the vaccine could potentially increase household willingness to pay for HepB (27). 20μg HepB-CHO was a self-pay vaccine, and 85.83% of children in our study received 20μg HepB-CHO at the Kecheng vaccination clinic, which was associated with improved attitudes and knowledge about vaccines in the main urban area of Kecheng district.

Our study had several limitations. First, we could not compare persistence in each group because antibody levels were not available at different time points. Second, the small sample size is a limitation of this study. With only about a hundred children in each group, it is difficult to generalize the results to the wider population.

In conclusion, the GMC and SPR of children four years after primary vaccination showed good immunogenicity, with the GMC of the 20 μg CHO Homo prime-boost group being higher than that of the 10 μg HP Homo prime-boost group. Almost 15% of the study sample were not protected and 5.01% had an anti-HBs concentration of ≤ 2.5 mIU/ml. Household willingness to pay for HepB could potentially be increased by working on attitudes and knowledge about the self-pay vaccine. Therefore, the booster doses were not necessary in the general children, the use of 20 μg HepB-CHO in the prime-boost would increase the anti-HBs concentration of primary vaccination, timely testing and improved knowledge about the self-pay vaccine would be good for controlling hepatitis B.

## Data availability statement

The raw data supporting the conclusions of this article will be made available by the authors, without undue reservation.

## Ethics statement

The studies involving humans were approved by the Ethics Committee of Quzhou Centers for Disease Control and Prevention (2021-R-NO.06). The studies were conducted in accordance with the local legislation and institutional requirements. Written informed consent for participation in this study was provided by the participants’ legal guardians/next of kin.

## Author contributions

ZY: Writing – review & editing, Writing – original draft, Project administration, Funding acquisition. TW: Writing – original draft, Formal analysis, Data curation. CF: Writing – review & editing, Visualization, Software. JL: Writing – review & editing, Supervision, Investigation. QF: Writing – review & editing, Supervision, Investigation. XG: Writing – review & editing, Supervision, Investigation. JY: Writing – review & editing, Validation, Investigation. SW: Writing – review & editing, Visualization, Software. CZ: Writing – review & editing, Supervision, Investigation, Data curation.
